# Gait Speed as a Marker of Superior Physical Performance in SuperAgers: Cognitive and Neuroanatomical Correlates

**DOI:** 10.1002/alz.088134

**Published:** 2025-01-03

**Authors:** Bori. R. Kim, Kee Hyung Park, Jee Hyang Jeong, Geon Ha Kim

**Affiliations:** ^1^ Department of Neurology, Ewha Womans University Mokdong Hospital, Ewha Womans University, College of Medicine, Seoul, Republic of Korea, Seoul Korea, Republic of (South); ^2^ Ewha Medical Research Institute, Ewha Womans University, Seoul, Korea, Seoul Korea, Republic of (South); ^3^ Gachon University Gil Medical Center, Gachon University College of Medicine, Incheon Korea, Republic of (South); ^4^ Ewha Womans University Seoul Hospital, Ewha Womans University College of Medicine, Seoul Korea, Republic of (South); ^5^ Ewha Womans University Mokdong Hospital, Ewha Womans University College of Medicine, Seoul Korea, Republic of (South)

## Abstract

**Background:**

Cognitive abilities, notably memory, typically decline with age, but a subset of individuals known as superagers defy this trend by exhibiting memory functions akin to those 20‐30 years younger in late life. Recognizing the interconnection between physical performance, health outcomes, and cognitive function in older adults, our aim was to explore whether superagers demonstrate superior physical performance compared to typical agers.

**Methods:**

Forty‐nine cognitively unimpaired older adults underwent comprehensive assessments, including cognitive function tests using the Seoul Neuropsychological Screening Battery, Brain MRI, and the Short Physical Performance Battery (SPPB), a prominent tool for evaluating physical function. Normal cognitive function was defined by scores above ‐1.0 SD of population norms for memory tests and above ‐1.5 SD for language, attention, visuospatial, and executive function tests. FreeSurfer software processed T1 images to obtain anatomical parcellations, including the hippocampus. The SPPB, consisting of standing balance, repeated chair stands, and gait speed measures, was conducted using an automated physical performance test instrument (AndanteFit, DYPHI Inc., South Korea). Superagers were identified based on their performance compared to middle‐aged adults (45 years old) in delayed recall scores from the Seoul Verbal Learning Test and the Rey‐Osterrieth Complex Figure test. Among the 49 participants, 26 were classified as superagers.

**Results:**

No significant differences in age (72.6 ± 7.4 vs. 71.2 ± 5.7, P = 0.465), sex (69.6% female vs. 80.8% female, P = 0.363), and education level (10.5 ± 4.2 vs. 11.1 ± 3.8, P = 0.582) were observed between typical agers and superagers. While total SPPB scores showed no significant difference, gait speed scores were notably higher in superagers (3.7 ± 0.6 vs. 4 ± 0, P = 0.008). Raw gait speed analysis confirmed a faster gait speed in superagers, and intriguingly, gait speed correlated significantly with memory function (r = 0.4182, P = 0.03) and left hippocampal volume (r = 0.6103, P = 0.04) in superagers.

**Conclusion:**

Our study suggests that superior physical performance, including gait speed, in superagers is associated with enhanced memory function and greater left hippocampal volume. This reinforces the importance of assessing physical function in clinical practice, particularly gait speed, as it may serve as a marker for cognitive health.

